# New Experimental Models of Retinal Degeneration for Screening Molecular Photochromic Ion Channel Blockers

**Published:** 2018

**Authors:** A. Yu. Rotov, L. A. Astakhova, V. S. Sitnikova, A. A. Evdokimov, V. M. Boitsov, M. V. Dubina, M. N. Ryazantsev, M. L. Firsov

**Affiliations:** Peter the Great St. Petersburg Polytechnic University, Polytekhnicheskaya Str. 29, St. Petersburg, 195251 , Russia; Sechenov Institute of Evolutionary Physiology and Biochemistry, Russian Academy of Sciences, Toreza Ave. 44, St. Petersburg, 194223, Russia; St. Petersburg State Chemical–Pharmaceutical Academy, Professor Popov Str. 14, St. Petersburg, 197376 , Russia; St. Petersburg National Research Academic University, Russian Academy of Sciences, Khlopina Str. 8/3A, St. Petersburg, 194021, Russia; St. Petersburg State University, Institute of Chemistry, Universitetskiy Ave. 26, St. Petersburg, Petergof, 198504, Russia; St. Petersburg National Research University of Information Technologies, Mechanics and Optics, Kronverkskiy Ave. 49, St. Petersburg, 197101, Russia

**Keywords:** photoreceptor degeneration, retinal degeneration, photochromic ion-channel blockers, photopharmacology, vision

## Abstract

Application of molecular photochromic ion channel blockers to recover the visual function of a degenerated retina is one of the promising trends in photopharmacology. To this day, several photochromic azobenzene-based compounds have been proposed and their functionality has been demonstrated on cell lines and knockout mouse models. Further advance necessitates testing of the physiological activity of a great number of new compounds. The goal of this study is to propose animal models of photoreceptor degeneration that are easier to obtain than knockout mouse models but include the main features required for testing the physiological activity of molecular photoswitches. Two amphibian-based models were proposed. The first model was obtained by mechanical deletion of the photoreceptor outer segments. The second model was obtained by intraocular injection of tunicamycin to induce the degeneration of rods and cones. To test our models, we used 2-[(4-{(E)-[4-(acryloylaminophenyl]diazenyl}phenyl)amino]-N,N,N-triethyl-2-oxoethanammonium chloride (AAQ), one of the compounds that have been studied in other physiological models. The electroretinograms recorded from our models before and after AAQ treatment are in agreement with the results obtained on knockout mouse models and reported in other studies. Hence, the proposed models can be used for primary screening of molecular photochromic ion channel blockers.

## INTRODUCTION

**Fig. 1:**
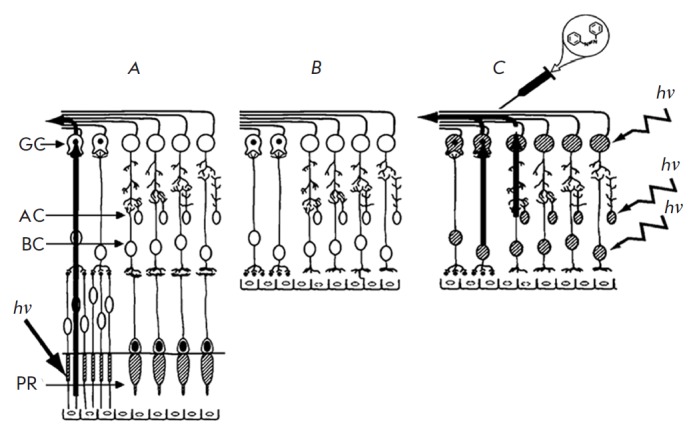
A schematic image of the retina. *A *– intact retina; *B *– retina with degenerated photoreceptors;* C *– retina with degenerated photoreceptors after the injection of photochromic compounds.* GC *– ganglion cells, *AC *– amacrine cells, *BC *– bipolar cells, *PR* – photoreceptors, *hv *– incident light. The light-sensitive cells are marked with hatching. Because the horizontal cells do not have direct access to the ganglion cells, they are not shown in the figure. The figure is adapted from ref. [3] (with changes)

Retinitis pigmentosa and age-related macular degeneration, which result in progressive degeneration of the retina, are widespread visual disorders [1, 2]. Progression of these disorders leads to the death of rods and cones, while other types of neurons in the retina – ganglion, amacrine, bipolar and horizontal cells – survive (*Fig. 1 A, B*). Transmission of information to the brain ceases due to the loss of photoreceptor cells; i.e., the visual function is lost.

To this day, no curative treatment for these diseases has been achieved. Therefore, new strategies aimed at visual restoration after complete photoreceptor degeneration are under active development. Several approaches in dealing with the problem have been proposed. For example, implanted electronic retinal prostheses have been shown to partially restore visual function in patients with total vision loss [4]. Transplantation of stem cells into the retina has restored reaction to light in blind mice [5]; transplantation of the retinal pigment epithelium has allowed to enhance vision in patients with age-related macular degeneration [6]. Another approach, the optogenetic one, implies incorporation of light-sensitive proteins into the retinal neurons using genetic engineering methods. These can be bacterial opsins (light-activated ion channels) or hybrid proteins containing light-sensitive domains of visual pigments and the C-terminal domains of metabotropic receptors that induce intracellular signaling. Both optogenetic methods lead to partial restoration of lightinduced processes [7, 8].

**Fig. 2:**
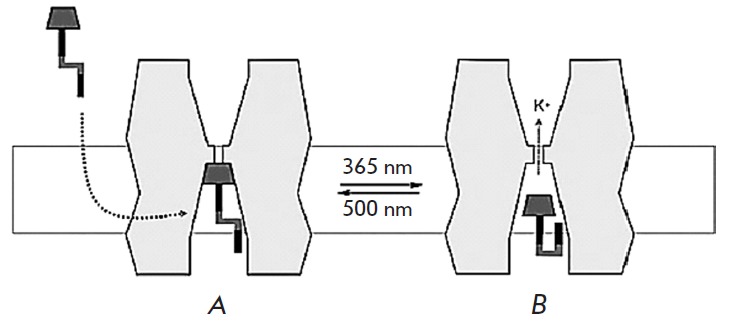
The mechanism of action of molecular photochromic compounds on K^+^ channels. *A *– the trans-form of the photochromic compound blocks the flow of ions through the channel, *B *– the cis-form does not prevent the flow of ions. The transition from one conformation to another takes place after light of a specific wavelength has been absorbed. The figure is adapted from ref. [9] (with changes)

However, all these approaches are characterized either by high invasiveness or by the irreversibility of adverse side effects. An alternative method for the restoration of the visual function of the retina with degenerated photoreceptors has been recently proposed. It involves the injection of photosensitive molecules into the retina, which can bind to the voltage-dependent potassium channels in the membrane of survived cells (ganglion, amacrine, bipolar and horizontal ones). However, the latter three types of cells have no direct access to ganglion cells; for this reason, they will not be further considered in this work. The bound molecule exists in the trans-form and blocks the ion current through the channel, until a photon with a specific wavelength is absorbed. The photochromic compound then isomerizes into its cis-form that does not block an ion channel, thus inducing an ionic current, changing the membrane potential, and generating a photoresponse (*Fig. 2*). The molecule isomerizes back into its stable trans-form either in darkness or under illumination with light of a longer wavelength [10]. Thus, the cells activated by the photochromic blocker start to respond to light stimulation in a specific range of wavelengths in order to restore afferent signaling from the eye to the brain (*Fig. 1C*).

The promise of such an approach has been demonstrated in electrophysiological experiments on HEK293 cells expressing potential-dependent potassium channels and on cultured hippocampal neurons [11]. In both cases, the cells acquired photosensitivity. Experiments with blind rd1 mice with a knockout Pde6b gene demonstrated a response to light stimulation in an isolated retina and a retina incubated with a photochromic compound and a change in the behavioral response to light in animals that had received an intraocular injection of the drug [12, 13].

Further research in this direction requires physiological screening of a large number of compounds to select the most effective ones. For this reason, an animal model of photoreceptor-specific retinal degeneration that is simpler than the model with knockout mice is required. We have created two new amphibian-based models. The first model was obtained by mechanical removal of its photoreceptor outer segments. In the second model, the degeneration of rods and cones was achieved by intraocular injection of antibiotic tunicamycin. Tunicamycin is known to disturb opsin glycosylation and the formation of membrane discs in outer photoreceptor segments, which leads to their degradation within 15–25 days [14, 15]. The advantage of this model over the mouse models mentioned above consists in the simplicity of manipulations with the retina of cold-blooded animals compared to warm-blooded ones.

Several models of photoreceptor degeneration in cold-blooded animals have been developed thus far, but all of them use fish [16], including the models that use tunicamycin injections. Yet, performing intravital control over retinal degeneration is more complicated for a fish-based model than it is for amphibian-based ones.

## EXPERIMENTAL

**Synthesis of acrylamide-azobenzenequaternary ammonium (AAQ, scheme)**

*4-[(E)-(4-Nitrophenyl)diazenyl]aniline (1a). *4-Nitroaniline (16.9 g, 0.12 mol) was partially dissolved in a mixture of water (50 mL) and concentrated aqueous HCl (50 mL) under heating in a water bath. The mixture was then cooled by pouring it into iced water, and a solution of sodium nitrite (8.4 g, 0.12 mol) in water (31 mL) was added under cooling on an ice bath. The resulting homogeneous mixture was stirred for 1 h; an ice-cold solution of aniline (11.4 g, 0.12 mmol) in a mixture of water (122 mL) and concentrated aqueous HCl (25 mL) was then added drop-wise for 30 min at 0–5 °C. After stirring in the ice-water bath for 2 h, the mixture was neutralized with aq. NH_3_. The resulting mixture was filtered and washed with water and ethanol. The red– brown powder was dried in vacuo to give (** 1a) **with a 66% yield. *Rf *= 0.74 (hexane–ethyl acetate 1:1 v/v). 1H NMR (400 MHz, CDCl3): δ 4.41 (Br. s, 2H), 6.63 (d, J = 8.1 Hz, 2H), 7.88 (d, J = 8.1 Hz, 2H), 8.08 (d, J = 8.7 Hz, 2H), 8.35 (d, J = 8.7 Hz, 2H).

*4,4’-(E)-Diazene-1,2-diyldianiline (1b). *A solution of amine (**1a) **(5.0 g, 18.5 mmol) and sodium sulfide nonahydrate (9.80 g, 37 mmol, 2 equiv) in ethanol (300 ml) was refluxed for 3 h. The solvent was evaporated under vacuum. The obtained brown oil was re-dissolved in a mixture of water (250 ml) and brine (15 ml). The product was extracted with ethyl acetate (3 x 100 ml), dried over magnesium sulfate, concentrated (to about 50 ml), and passed through a short pad of silica gel (25 g). Compound **(1b) **(3.2 g, 80%) was obtained as a reddish solid (mp. 234-237°C). *Rf *= 0.55 (hexane–ethyl acetate 1:1 v/v). 1H NMR (400 MHz, DMSO-*d*6) δ 5.73 (s, 4H), 6.61 (d, J = 8.7 Hz, 4H), 7.52 (d, J = 8.7 Hz, 4H). 13C NMR (100 MHz, DMSO-*d*6) δ 113.3, 124.2, 142.8, 151.4.

*N-{4-[(E)-(4-Aminophenyl)diazenyl]phenyl}acrylamide (2a) and N,N’-[(E)-diazene-1,2-diylbis(4,1-phenylene)] bisacrylamide (2b). *A solution of acryloyl chloride in dichloromethane (0.38 ml, 4.7 mmol) was added dropwise to a solution of amine **(1b) **(1.0 g) in dichloromethane (200 ml) at 0°C. The obtained reaction mixture was stirred at room temperature for 12 h. After that, the solvent was evaporated. The products were analyzed by thin-layer chromatography (TLC) and then separated by column chromatography using silica with hexane: ethyl acetate as an eluent.

Compound **(2a)**: orange-red solid (19%); 1H NMR (400 MHz, DMSO-*d*6): 4.72 (s, 2H), 5.78 (dd, *J *= 10 and 2 Hz, 1H), 6.31 (dd, *J *= 17 and 2 Hz, 1H), 6.47 (dd, *J *= 17 and 10 Hz, 1H), 7.65 (d, *J *= 8.7, 2H), 7.79-7.94 (m, 6H), 10.34 (s, 1H). ^13^C NMR (100 MHz, DMSO-*d*_6_): 114.6 (2C), 121.3 (2C), 124.2 (2C), 125.7, 127.9 (2C), 132.4, 141.3, 145.3, 149.9, 152.5, 164.5. HRMS (ESI): *m/z *[M + H]^+^ calcd for C_15_H_16_N_4_O: 268.1319, found: 268.1324.

Compound **(2b)**: brown-red solid (42%); ^1^H NMR (400 MHz, DMSO-*d*_6_): 5.82 (dd, *J *= 10 and 2 Hz, 2H), 6.33 (dd, *J *= 17 and 2 Hz, 2H), 6.50 (dd, *J *= 17 and 10 Hz, 2H), 7.85–7.93 (m, 8H), 10.51 (s, 1H). HRMS (ESI):* m/z *[M + H]^+^ calcd for C_18_H_18_N_4_O_2_: 322.1424, found: 322.1431.

*N-[4-((E)-{4-[(2-Chloroacetyl)amino]phenyl}diazenyl) phenyl]acrylamide (3). *A solution of chloroacetyl chloride (0.17 ml, 1.2 equiv.) in dichloromethane (5 ml) was added to a vigorously stirred solution of amine **(2a)** (1.8 mmol, 0.5 g) and DIPEA (0.9 ml, 3 equiv.) in dichloromethane (15 ml) at 0°C. The reaction mixture was stirred at room temperature for 12 h, quenched with water, and mixed with dichloromethane (2 × 20 ml). The combined organic layers were washed with a 5% aqueous HCl solution (15 ml) and water (15 ml), dried over sodium sulfate, and evaporated. The crude product was purified by flash chromatography using silica with hexane-ethyl acetate as an eluent to give rise to** (3) **as a red solid with a 53% yield. 1H NMR (400 MHz, DMSO-*d*_6_): δ 4.32 (s, 2H), 5.83 (dd, *J *= 10 and 2 Hz, 1H), 6.32 (dd, *J *= 17 and 2 Hz, 1H), 6.50 (dd, *J *= 17 and 10 Hz, 1H), 7.81–7.90 (m, 8H), 10.48 (s, 1H), 10.64 (s, 1H). HRMS (ESI): *m/z *[M + H]^+^ calcd for C_17_H_17_ClN_4_O_2_: 344.1035, found: 344.1031.

*2-[(4-{(E)-[4-(acryloylaminophenyl]diazenyl}phenyl) amino]-N,N,N-triethyl-2-oxoethanammonium chloride (4, AAQ). *Triethylamine (0.2 mL) was added to the solution of compound **(3) **(0.3 g, 0.9 mmol) in dimethylformamide (5.0 mL). The mixture was then heated for 12 h at 50°C in a nitrogen atmosphere. The reaction mixture was cooled to room temperature, and the solvent was removed in vacuo. The resulting red solid was dissolved in distilled water, and the insoluble precipitate was filtered off. Water was removed under vacuum to obtain pure compound **(4) **as a red solid with a 45% yield. ^1^H NMR (400 MHz, D_2_O): δ 1.12 (t, *J *= 7 Hz, 9H), 3.37 (m, 6H), 4.35 (s, 2H), 5.77 (dd, *J *= 10 and 2 Hz, 1H), 6.29 (dd,* J *= 17 and 2 Hz, 1H), 6.47 (dd, *J *= 17 and 10 Hz, 1H), 7.69–7.75 (m, 4H), 7.82-7.89 (m, 4H), 11.71 (s, 1H), 12.11 (s, 1H). 13C NMR (100 MHz, D2O): 7.8 (3C), 53.3, 56.5 (3C), 120.5 (2C), 121.1 (2C), 123.2, 125.2 (2C), 128.8 (2C), 132.8, 141.8, 142.6, 148.5, 149.7, 160.4, 163.9. HRMS (ESI): *m/z* [M – Cl]^+^ calcd for C_23_H_31_N_5_O_2_: 409.2472, found: 409.2469.

**The models of photoreceptor degeneration in cold-blooded animals**

Marsh frogs (*Rana ridibunda*) caught in the Astrakhan Region of Russia were used in our experiments. The animals were kept in the vivarium of the Institute at 20°C and 12/12 light cycle; they were fed flour worms.

**The model with mechanical removal of photoreceptors**

First, the method of mechanical removal of the photoreceptor layer from the retina was tested. The retina extracted from the eyecup was placed on a filter paper sheet with the photoreceptors on the paper surface. The outer segments of the photoreceptors are connected to the inner segment layers by a thin cilium that can be easily broken. Thus, we just needed to remove the paper with the photoreceptor layer on it to obtain a retina model devoid of light-sensitive outer photoreceptor segments.

The obtained model was tested in electrophysiological experiments. The preparation was placed in a chamber filled with an amphibian Ringer solution. Two identical silver/silver chloride electrodes (World Precision Instruments, Inc., USA) in contact with the medium from different sides of the retina were used for transretinal ERG recording. The composition of the Ringer solution for preparations of the isolated retina was as follows: 90 mM NaCl, 2.5 mM KCl, 1.4 mM MgCl_2_, 10 mM glucose, 1.05 mM CaCl_2_, 5 mM NaHCO_3_, 5 mM HEPES, 0.05 mM EDTA, and 50 mg/l bovine serum albumin. White (415–745 nm) and ultraviolet (UV, 365 nm) LEDs were used for stimulation. Signal intensity was controlled by changing the electric current flowing through the LEDs and by using neutral-density filters. ERG was recorded with 5 ms sampling for each point with analogous filtration in the 0–100 Hz band using an 8-pole Bessel filter. The experimental setup controlling program was custom written in the laboratory using Microsoft Visual Basic 96.

**Fig. 3:**
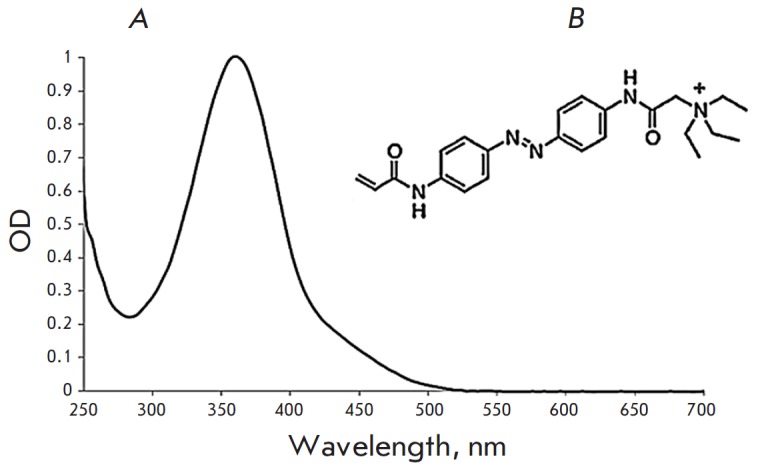
*A *– spectrum of the trans-form of AAQ in the amphibian Ringer solution; *B *– the structural formula of AAQ

The model of the retina without photoreceptors was tested using a AAQ photochromic compound whose efficiency was known from the study performed on knockout mice [12]. The retina was placed in a chamber filled a witha 1 mM AAQ solution (the Ringer solution was used as a solvent) and incubated for 30 min. The chamber containing the retina sample was then filled with a pure Ringer solution, and ERG was recorded. AAQ is known to change conformation under illumination with a 365-nm wavelength (*Fig. 3*). For this reason, the sample was tested using brief flashes and long exposures to UV LED. Green light was used as the control signal (520 nm, white LED with an appropriate color filter).

**The model of tunicamycin-induced photoreceptor degeneration**

A tunicamycin solution (Sigma, 1 μg of the antibiotic per 10 g of frog weight) in DMSO at a concentration of 0.5 mg/ml was injected into one eye of the frogs. The second eye was used as the control; the same volume of pure DMSO was injected into it. *In vivo *ERG was recorded 2 h after the injection and then subsequently with a 7-day interval. Before the injections and ERG recordings, the frogs were anesthetized with a MS-222 drug (Sigma, 1 mg/ml aqueous solution). Recording from the surface of the eye cornea was performed using the same experimental setup as the one used for ERG recordings from the isolated retina. To achieve such recordings, a silver wire electrode that was in contact with the cornea through a conductive ophthalmic gel was used; a white diffuser ensuring a uniform distribution of light over the pupil area was employed. A silver wire electrode inserted into the oral cavity of the animal was used as the control electrode. Light stimulation was performed with short (10 ms), white LED flashes. Thus, the entire process of photoreceptor degeneration was monitored. After the photoresponse in animals had disappeared, they were decapitated and a preparation of the retina devoid of photoreceptors was obtained. The parameters (sampling and the filtering band) were the same as those used during ERG recording from the isolated retina with mechanically removed photoreceptors.

The retina preparation extracted from the eye that had been exposed to tunicamycin and lost photosensitivity was additionally tested for lack of ERG in Ringer solution and then in a the presence of a photochromic compound, AAQ, in the same manner as the model with mechanically removed photoreceptors.

**Morphological control over photoreceptor degeneration**

After the photoresponse in the tunicamycin-exposed eye had disappeared, the eyecups of three decapitated frogs were taken for the subsequent histological investigation. The fixation protocol was the same as described by Sillman et al. [18]. The eyecups were placed into a 1% glutaraldehyde solution in 0.1 M phosphate buffer for 1.5 h. The preparations were then rinsed with a 4% paraformaldehyde solution in 0.1 M phosphate buffer for 4 h and kept in a 1% paraformaldehyde solution for several weeks. The preparations were then washed with 0.1 M phosphate buffer, dehydrated with ethanol, and fixed in a LR White epoxy resin (Fluka). Slices (1–3 μm thick) were prepared using an LKB ultramicrotome, stained with toluidine blue, and assessed by light microscopy to identify the layer of photoreceptor cells.

## RESULTS AND DISCUSSION

**Synthesis of acrylamide-azobenzenequaternary ammonium (AAQ)**

Water-soluble acrylamide-azobenzene-quaternary ammonium was synthesized in several simple stages from simple and easily accessible compounds. The azo group was inserted via the reaction of the corresponding diazonium salt with aniline (*Scheme*). The obtained 4-nitroazobenzene **(1a)** was reduced to 4,4’-diaminoazobenzene **(1b)** with sodium sulfide in boiling ethanol (*Scheme*). Monosubstituted 4,4’-diamonoazobenzene** (2a) **was obtained via the reaction between azobenzene** (1b) **and acrylic acid chloride. The corresponding diadduct was formed as a by-product of this reaction **(2b)** (*Scheme*). A quaternary ammonium cation was inserted into the target product in two stages (*Scheme*). The amide **(3) **was produced in the reaction between aminoazobenzene **(2a) **and chloroacetic acid. The reaction between the resulting chloroacetyl chloride and triethylamine gave rise to target compound** (4), (AAQ)** with moderate yields.

**Photoreceptor degeneration models in cold-blooded animals**

**Fig. 4:**
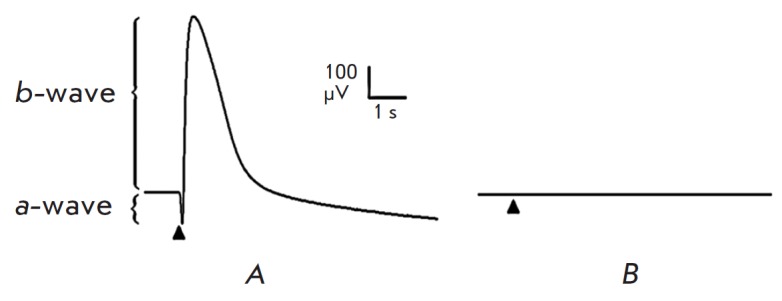
*A *– ERG of the intact retina preparation in response to light stimulation. *B *– ERG of the retina preparation after mechanical removal of photoreceptors. Stimulation was performed with green light (520 nm; duration of the flash, 10 ms; flash intensity, 2.9 × 10^6^ photons/μm2/s). The moment of flash is shown with a triangle

**Fig. 5:**
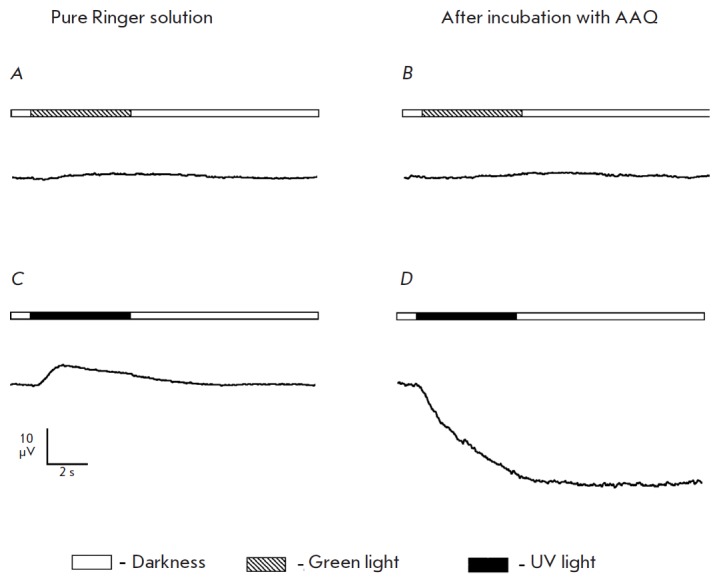
Comparison of the trans-retinal ERG of retina with mechanically removed photoreceptors that occurs in response to green and UV light stimuli in a pure Ringer solution (*A,C*) and after 30 min of incubation in 1 mM AAQ (*B,D*). The scheme of stimulation is given for each plot. The stimulation duration is 5 s. The intensity of green light (520 nm) is 2.9 × 10^8^ photons/ μm^2^/s; the intensity of UV light (365 nm), 6.5 × 10^8^ photons/μm^2^/s

*The model with mechanical removal of photoreceptors.* An ERG of the intact retina sample contains both expected components: a-wave characterizing the hyperpolarization of photoreceptors and b-wave arising from the function of Muller cells and bipolar cells (*Fig. 4A*). Either a lack of response to UV and visible light stimulation or weak residual responses with an amplitude of several μV is observed after the photoreceptor layer has been mechanically removed (*Fig. 4*). The residual response retains only the b-wave, while the a-wave completely disappears (*Fig. 5 A, C*). These results demonstrate that the employed approach allows one to obtain a working model of retinal degeneration which can be used in further studies.

**Fig. 6:**
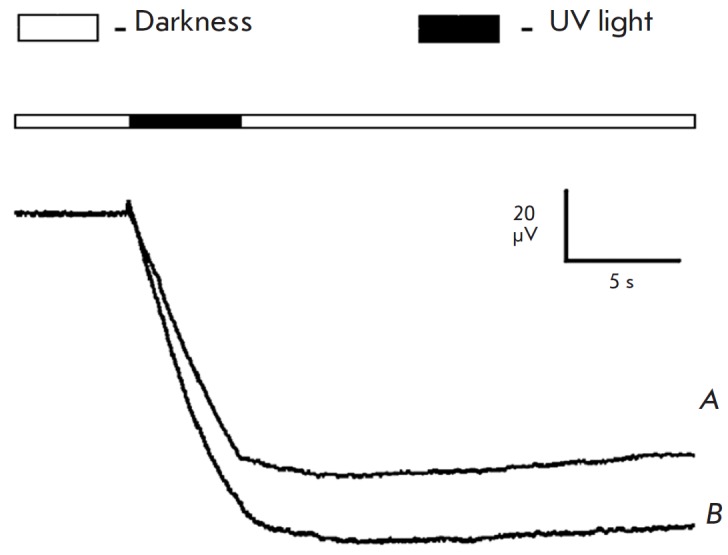
The response of the retina with mechanically removed photoreceptors to long-term (5 s) UV illumination (365 nm) of differing intensity after incubation with AAQ.* A – *intensity, 2.7 × 10^8^ photons/μm^2^/s, *B – *intensity, 6.5 × 10^8^ photons/μm^2^/s

*Results of incubation in AAQ solution. *As expected, stimulation of a retina sample after its incubation in the AAQ solution with green light led to naught response (*Fig. 5B*). No response to brief flashes of UV light oc curred, while long-term UV light stimulation led to a response pointed in the same direction as a normal ERG a-wave with an amplitude ranging from 10 to 100 μV (*Fig. 5D*). This response ceased right after the illumination had been turned off. The potential had either never returned to its initial value or the process was very slow. The amplitude of the occurring response depended on the intensity of the light stimuli: the higher the intensity of the UV stimuli, the larger the change in the potential was (*Fig. 6*).

**Scheme:**
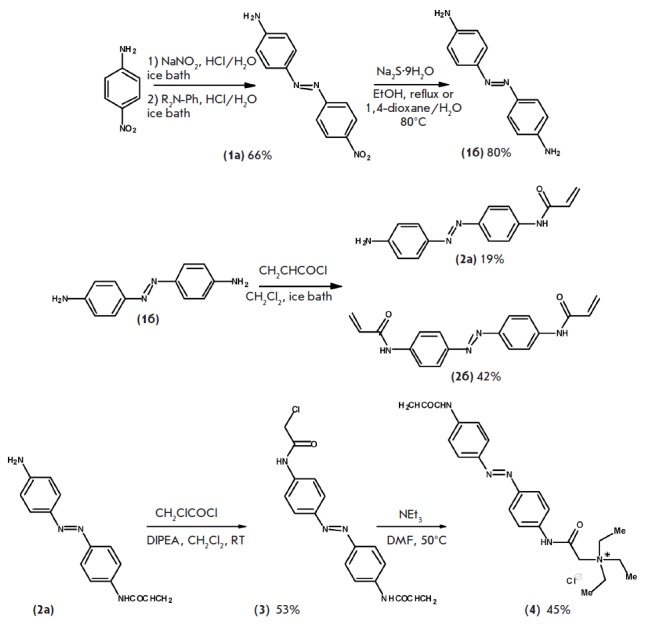
Synthesis of acrylamide-azobenzene-quaternary ammonium

**The model of tunicamycin-induced photoreceptor degeneration**

**Fig. 7:**
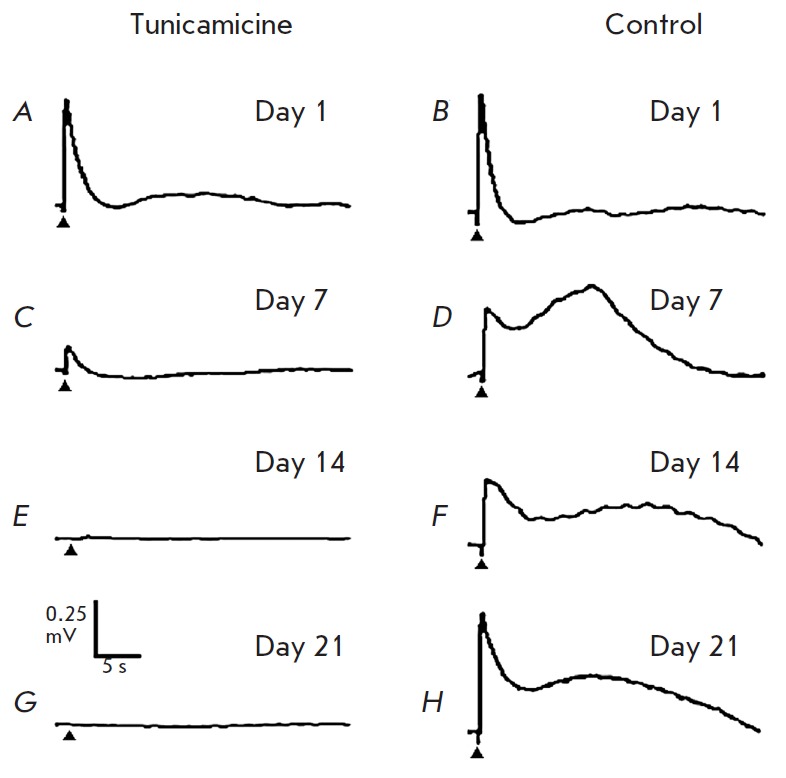
ERG registered from the cornea of an anesthetized frog at different periods of time post-injection. *A, C, E, G *– from the eye into which tunicamycin was injected; *B, D, F, H *– from the eye into which only DMSO was injected. Stimulation with white light (range, 415–745 nm); flash duration, 10 ms; intensity, ~9 W/μm^2^ of the pupil area. The moment of flash is shown with a triangle

Injections of tunicamycin led to a progressing decrease in the response amplitude to brief flashes of light (*Fig. 7*). As one can see in *Fig. 7, panel a*, the ERG amplitude for the eye with tunicamycin injection gradually decreased and completely disappeared on days 14–21. On the contrary, the ERG of the control eye into which a pure solvent (DMSO) was injected (*Fig. 7, panel b*) did not change.

**Fig. 8:**
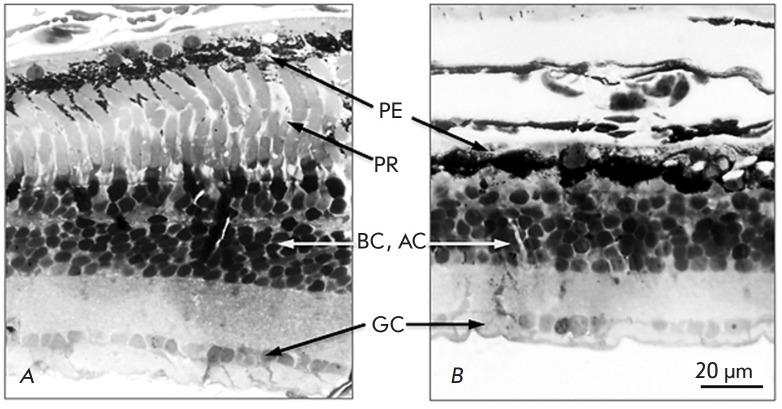
Light microscopy images of retina preparations. *A *– the retina of the control eye into which DMSO was injected.* B *– the retina of the eye into which tunicamycin was injected. *PE *– pigment epithelium,* GC *– ganglionic cells,* AC *– amacrine cells, *BC *– bipolar cells, *PR *– photoreceptors. The photoreceptor layer is absent, but other cell types remained in the retina exposed to tunicamycin

**Fig. 9:**
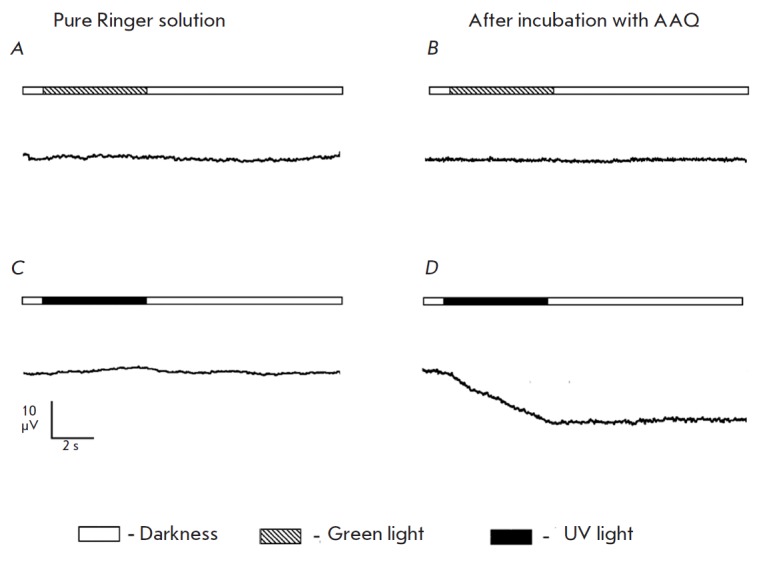
Comparison of the transretinal ERG of the retina degenerated after being exposed to tunicamycin, which occurs in response to illumination with green and UV light in a pure Ringer solution (*A, C*) and after 30 min of incubation in 1 mM AAQ (*B, D*). The scheme of light stimulation is given for each diagram. Duration of each light stimulation is 5 s. The intensity of green light (520 nm) is 2.9 × 10^8^ photons/ μm^2^/s; the intensity of UV light (365 nm) is 6.5 × 10^8^ photons/μm^2^/s

Microscopy of the eyecup slices confirmed that exposure to tunicamycin caused selective degeneration of photoreceptor cells. In *Fig. 8A* showing a transverse section of the eyecup from the control eye (DMSO injection), all retinal layers are well-distinguishable. In the retina of the eye exposed to tunicamycin, the layer of photoreceptor cells is absent (*Fig. 8B*) but bipolar, amacrine, and ganglion cells remain. Therefore, such a degeneration model can be used to test molecular photochromic compounds. The isolated retina of the eye exposed to tunicamycin responded to stimulation with neither green nor UV light (*Fig. 9 A,C*), which is consistent with the results of *in vivo *ERG recordings and shows that the performed manipulations yielded the desired result: a model of photoreceptor degeneration was obtained.

**Results of incubation in AAQ solution**

Incubation in the AAQ solution induced a response to long-term UV illumination unidirectional to that of a normal ERG a-wave, with an amplitude of 10–30 μV (*Fig. 9 B,D*), similar to the model of retina with mechanically removed photoreceptors. The signal recorded after the illumination was turned off remained stable, thus indicating that the nature of the response is identical to that of the model with mechanically removed photoreceptors. Stimulation with green light did not lead to any response.

The results show that AAQ restores photosensitivity to a model preparation in the near-UV region, possibly via light-dependent regulation of ion channels by the photochromic compound. Both models of degenerated retina yield similar results.

## CONCLUSIONS

In this study, we have introduced two experimental models of photoreceptor degeneration that can be used for the primary screening of new molecular photochromic potassium channel blockers.

**Fig. 10:**
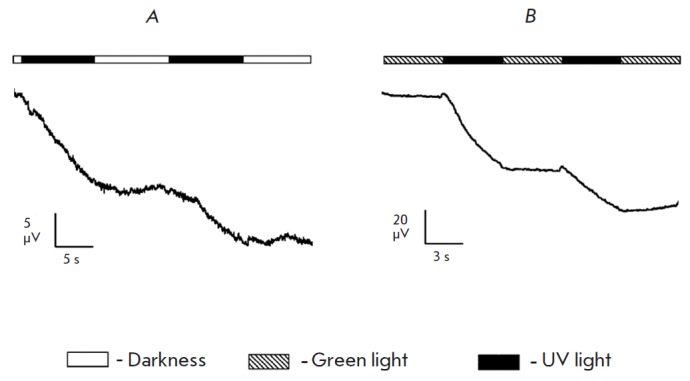
*A *–ERG of the retina degenerated after being exposed to tunicamycin under alteration of UV illumination (365 nm) and dark cycles after 30 min of incubation in 1 mM AAQ. B – ERG of the retina with mechanically removed photoreceptors under alteration of illumination with UV (365 nm) and green light (520 nm) after 30 min of incubation in 1 mM AAQ. The scheme of light stimulation is given for each diagram. The intensity of green light is 2.9 × 10^8^ photons/ μm^2^/s; the intensity of UV light is 6.5 × 10^8^ photons/μm^2^/s

The findings on the effect of AAQ on ERG obtained in this study show good agreement with the results reported by other authors who demonstrated that this compound is able to impart photosensitivity in the UV region to the degenerated retina [12]. The effect of molecular photochromic compounds demonstrated by the authors was as follows: the frequency of spike generation by ganglion cells increased significantly under long-term UV stimulation (a multi-electrode array was used for recording), while returning to the initial level after the light had been turned off or replaced with green light. We have demonstrated in this study that a different pattern is observed upon alternation of UV stimulus and a dark cycle or a UV stimulus and green light: the potential changes only during exposure to UV, while signal intensity remains stable under dark conditions or under illumination with green light but does not return to its initial level (*Fig. 10*).

Our data also indicate that AAQ cannot be regarded as a substance for vision restoration to be used in clinical practice not only because it is unable to function under light visible to the human eye, but also due to its ultra-slow response to a light stimulus. However, the amphibian-based models of photoreceptor degeneration proposed in this work can be used to test new compounds and identify the most promising ones. The approach with mechanical photoreceptor removal allows one to quickly obtain a model preparation; therefore, its application accelerates the screening of photochromic compounds. On the contrary, tunicamycin-induced photoreceptor degeneration is a gradual process and the changes inherent to common retina-degeneration diseases, such as retinitis pigmentosa or age-related macular degeneration, have time to occur [19]. There fore, the tunicamycin model allows one to study the action of molecular photochromic compounds on a model that is closer to a degenerated retina.

The model of tunicamycin degeneration can be further tested using laboratory rats in order to obtain a model that would be more valid for humans and to investigate the effect of these compounds on the retina of warm-blooded animals. Attempts to create a model of tunicamycin photoreceptor degeneration on rats have already been reported [20], but the action of molecular photochromic compounds under this model has not been studied yet.

## Abbreviations

AAQ: 2-[(4-{(E)-[4-acryloylaminophenyl]diazenyl}phenyl)amino]-N,N,N-triethyl-2-oxoethaneammonium chloride acrylamide-azobenzene-quaternary ammonium
ERG: electroretinogram
